# Fostering health professional students’ wellbeing during COVID -19 lockdown

**DOI:** 10.15694/mep.2020.000248.1

**Published:** 2020-11-06

**Authors:** Farzana Mahdi, Sonia Jaiswal, Sucheta Dandekar

**Affiliations:** 1 Era's Lucknow Medical College

**Keywords:** COVID-19, wellbeing, physical, emotional, spiritual, social, intellectual, environmental, occupational, mentorship

## Abstract

This article was migrated. The article was marked as recommended.

The unprecedented lockdown caused by the COVID-19 pandemic has resulted in an academic frenzy for students and teachers alike. Medical schools have had to take charge of the situation. Distance learning has become the norm. At the same time, online assessment is being considered to be rolled out to facilitate learning. Students went home during the lockdown and online classes were conducted for them to avoid any compromise to their learning. Facilitators were concerned about the students’ wellbeing as lack of personal contact made it difficult to assess their wellness. The virtual platform can be used as a tool to check the overall health status of the students. Well- being can be discussed in terms of physical, temperamental, cognitive and spiritual health along with their awareness towards the environment, social and professional attitudes. The help of a mentorship program can be sought to get in touch with students. Meetings can be set up with the mentees to help ease out the stress and worry from the student’s lives. This article attempts to suggest ways in which the students’ contentment can be met with in these trying times. The unprecedented lockdown caused by the COVID-19 pandemic has resulted in an academic frenzy for students and teachers alike. Medical schools have had to take charge of the situation. Distance learning has become the norm. At the same time, online assessment is being considered to be rolled out to facilitate learning. Students went home during the lockdown and online classes were conducted for them to avoid any compromise to their learning. Facilitators were concerned about the students’ wellbeing as lack of personal contact made it difficult to assess their wellness. The virtual platform can be used as a tool to check the overall health status of the students. Well- being can be discussed in terms of physical, temperamental, cognitive and spiritual health along with their awareness towards the environment, social and professional attitudes. The help of a mentorship program can be sought to get in touch with students. Meetings can be set up with the mentees to help ease out the stress and worry from the student’s lives. This article attempts to suggest ways in which the students’ contentment can be met with in these trying times.

## Introduction

COVID-19 pandemic resulted in a radical shift across the globe in terms of teaching and training in schools and universities. This sudden transition has added to the discomfiture of students and facilitators alike. Medical schools in India have also had to face the brunt. Since the students have been at the receiving end, analysing their wellbeing during this period of turmoil is an issue that needs active consideration.

Wellbeing is a concept that encompasses a positive and happy life. Apart from the material aspects, wellbeing is nurturing healthy relationships, realizing one’s own potential and having a positive outlook towards life ( Diener and Seligman, 2004; Diener, Napa and Lucas, 2009). In 1948, the WHO stated that being healthy encompasses physical, mental and social health apart from being free from all kinds of diseases ( Grad, 2002). These concepts have evolved over a period of time ( Hettler, 1984) to help not only academic education but also other businesses and programs to correlate between personal and professional values ( Roslender, Stevenson and Kahn, 2006; Brett and Cathleen, 2009).

The dimensions of wellbeing include physical wellbeing, which focuses on lifestyle choices and physical health ( Myers and Willard, 2003). Spiritual wellbeing revolves around a belief which is larger than “self”’ ( Hettler, 1984). Intellectual wellness is all about the stimulation of the mind. Managing reliable steadiness of temperament is also known as emotional health wherein a person is not only aware of positive and negative feelings but also strives towards stress reduction ( Adams, Bezner and Steinhardt, 1997). The ability and willingness to give support to others is social wellbeing ( Allison, 1997). Environmental wellness includes a relation to one’s surroundings ( Ghiselli, La Lopa and Bai, 2001). Occupational wellbeing links job satisfaction and life satisfaction ( Morgeson and Humphrey, 2006). This entire concept of wellness needs to be understood and integrated into an institution in order to make it an asset.

As faculty teaching in a medical school, we lack the insight of what can be done to find out how our students are faring especially in the light of the online classes, internet issues and other problems. We know that the long hours in front of the electronic devices have taken a toll of the health of students ( O’Doherty *et al*., 2018) and the aspect of well-being is ignored by most. Therefore, the seven aspects of well-being can be used as scaffolding on which we can consolidate our remote relationships. Mentoring is a valuable tool which gives emotional support to the students. The prevalence and positive effect of mentoring has generated a lot of interest in enthusiastic facilitators as it motivates the students towards setting their educational, professional and personal goals ( Guyatt, Feeny and Patrick, 1993). The students have to learn to achieve these goals during the lockdown period through technology, using synchronous and asynchronous interactions with their facilitators. Thus, mentorship programs shall go a long way in establishing a relationship of trust.

We present to you a way in which different dimensions can be used to ensure the students’ wellbeing during these trying COVID-19 pandemic times. Some aspects considered here, may be specific to certain socioeconomic groups. However, we are sure the exasperation felt towards the situation we are currently facing is the same globally and hope that all health professionals will be able to adapt to these as per individual needs.

## Tenets of Well-being

### Enhancing physical health

The scope of physical health is often associated with physical fitness wherein the body can perform various tasks efficiently and is “disease free”. The physical aspect of healthfulness helps in achieving independence. Certain lifestyle choices can help in maintaining or improving health and functional ability. The quality of life is a multifaceted concept which should be seen as a lifetime commitment ( Slavin, Schindler and Chibnall, 2014). It is indeed challenging for students to evaluate their own health status. As responsible faculty, inquiry about physical health is a necessity. Incorrect posture and physical inactivity can take a toll on brain health and cognition. Yoga lessons are given to the students in India, when they join the medical course. Whether they practice it regularly or not can be questioned. Physical well- being can be taken care of with the help of a balanced diet, exercise on a regular basis and rejuvenating the body through proper sleep. As a reminder, links to some exercise and yoga schedules can be sent to the students.

### Moving towards optimism

People with emotional wellness are confident and take charge of their feelings and behaviours. Students can become more optimistic, confident and develop a sense of self-regard ( Adam, Bezner and Steinhardt, 1997). Therefore, emotional wellbeing is more than an absence of disease since emotional distress causes susceptibility to physical illness. Managing emotions can be different for introverts and extroverts as categorized by the Meyer Brigg’s model ( Fylkesnes and Ford, 1991). Whether the lockdown is pushing students towards isolation can be asked. At the same time do they need a virtual platform for interactions with their mentors? Helping students understand their positive and negative feelings will help them to improve their emotional status. A virtual mentorship platform can be developed so that students can keep their emotional stress at bay. Meditation techniques, reading, going through inspirational quotes and animated discussions with their mentors can solve many problems.

### Expansion of knowledge and skills

Paying attention to one’s own intellectual dimension helps in increasing concentration skills, memory and critical thinking. Intellectual wellness is a stimulation of the mind, which can improve by engaging oneself in meaningful conversations. Fostering intellectual wellness cultivates mental growth. Intellectual perception changes behaviour and thereby becomes an asset for an institution ( Roslender, Stevenson and Kahn 2006; Hale, Hannum and Espelage, 2005). Several intellectual impacts surfaced during the lockdown crisis when the online classrooms took over the traditional classroom setting and teaching material was shared via mails. The teachers can show videos, encourage communication with relatives as simulated patients and actively discuss the situation with some hands-on learning of skills to address the problem of remote learning. Online internal assessments can be carried out with constructive feedback to gauge the learning. A virtual platform for student feedback can be developed so that the students can voice their concerns. The students can be asked to follow a disciplined routine with an organized weekly timetable which will help them to complete not only their studies but also other chores in hand. The mentors can encourage students to talk about their hobbies as pursuing hobbies stimulates the intellectual components.

### Building healthy relationships

Social wellness is about connectedness and a sense of belonging. It involves setting healthy boundaries, being assertive rather than passive or aggressive and using good communication skills. A healthy balance must be maintained to maximise the potential for personal wellbeing ( Chandler, Holden and Kolander, 1992). The social distancing has affected the students in many ways which includes missing group studies and social interactions with peers. The problem can be addressed with the help of a social electronic platform which provides continuous and effective communication. Students being technology friendly can adapt quickly to the virtual classroom interactions shedding their initial inhibitions.

Nonetheless the students are now appreciating family time available to them. Those with grandparents staying with them can be encouraged to look after their health. After all they are health professionals in the making! Phone calls to the elderly relatives and neighbours, to ask about their health are another means of keeping in touch with the community.

### Harmony with inner self and outside world

Spiritual wellness may come from activities such as reaching out to people, introspection, meditation, invocation, and appreciating nature. A strong spiritual health includes having clear core values, a sense of integrity and self-confidence, and a feeling of inner mental peace. It is an ability to construct a deeper meaning about life and is believed to be more fundamental than the other dimensions ( De Klerk, 2005). Deeper the understanding of spiritual wellness or energy, and development of spiritual purpose, the higher the level of an individual’s own wellness ( Swarbick, 2006; Adams, Bezner and Steinhardt, 2000; Kitko, 2001). Facilitators can encourage students to relax and take up activities such as volunteering to help the needy which can bring about a sense of connect with the inner self. Meditation and yoga are encouraged in India. Students belonging to diverse cultures and religions can be asked how they celebrated the festivals during lockdown. This can be handled by the mentors and the students can be greeted on such occasions.

### Preserving the surrounding

Social and natural surroundings have a great impact on one’s health. Cluttered environment can adversely affect one’s behaviour or perception ( Lengfelder, 1987). Pollution, violence, garbage build up, and water scarcity are some of the factors affecting environmental wellness. Facilitators can show concern for students who stay in remote areas and are inaccessible due to network problems. Reaching out to them and ensuring the comfort of the surroundings they stay in, are of paramount importance and should be done with vigour. A conscientious effort to make the environment better must be made ( Ureda and Yates, 2005). The students can be encouraged to switch off apps which distract them and create a space in time dedicated to studies.

### Finding satisfaction and meaning through work

Occupational wellness inspires us to work hard. One can find personal satisfaction and enrichment in life with the right kind of attitude towards work. The route to occupational wellness is to discover new opportunities and develop skills that are personally and professionally rewarding. A student’s occupational success is not merely dependent on the time spent in acquiring knowledge in the classroom but fostering other dimensions of wellness will also help in their growth and development as thorough professionals ( Dell Fave and Massimini, 2003; Dorn, 1992). The facilitators can ensure that there is no dearth of study material through virtual platforms. Hands -on experiences can be provided by telemedicine. Health professionals’ education being incomplete without the physical contact with patients, they can apprehensive about their future. This has to be dealt by active counselling measures. Reiteration that nothing is permanent and this too shall pass should be the motto. Some essential teaching components can be repeated when things are back to normal.

## Conclusion

This period of crisis may seem daunting but a little change in perception towards the crisis may help to bring about a state of total wellbeing. It can be observed that there is no clear demarcation between the different dimensions and they merge into one another, thus contributing towards making of a wholesome individual. Innovative methods may be sought to stay connected with the students and adaptations can be made as per the individual situations. There is, no doubt, a necessity to stay connected with the core values, following a disciplined schedule and de- cluttering our minds every now and then to suit the present situation.

*“The body achieves what the mind believes”* - *Napoleon Hill*

## Take Home Messages

• The lockdown era due to the COVID-19 pandemic can be treated as a period of introspection and innovation.
• Health is a state of wellbeing which encompasses all dimensions of wellness.
• Technology when combined with human emotions can delimit distances.
• A committed mentor can shape a mentee’s future.

## Notes On Contributors

**Dr. Farzana Mahdi**, is currently the Vice Chancellor of Era University and the Director Academics of Era’s Lucknow Medical college. She completed her FAIMER fellowship from GSMC, Mumbai. She has made numerous presentations in various national and international conferences in the field of medical education. Her latest venture is to exploit molecular tools and techniques to establish a new healthcare approach based on personalised medicine for the betterment of the society. ORCID ID: https://orcid.org/0000-0002-7955-7914

**Dr Sonia Jaiswal**, is currently working as an Assistant Professor in the Department of Anatomy at Era’s Lucknow Medical College. She graduated from King George Medical College and has received many awards during her graduation. Dr Sonia Jaiswal is a PhD in Anatomy and an avid researcher. Dr Sonia Jaiswal is an active member of the medical education department at Era’s Lucknow Medical College. She is the resource person for skill lab training. ORCID ID: https://orcid.org/0000-0003-2291-6178

**Dr. Sucheta Dandekar**, was Professor and Head of the Department of Biochemistry at the Seth G.S. Medical College and K.E.M Hospital, Parel, Mumbai. She is currently, Professor of Biochemistry at Era’s Lucknow Medical College. She is an adjunct faculty at the Manipal Academy of Health Education. Dr Dandekar is an active member of the GSMC-FAIMER and PSG - FAIMER regional institutes. She has been a keynote speaker at many conferences and has conducted numerous workshops in medical education. ORCID ID: https://orcid.org/0000-0001-5287-7160

## Declarations

The author has declared that there are no conflicts of interest.

## Ethics Statement

This manuscript includes the perspectives of the authors, and does not involve any investigation of human subjects.

## External Funding

This article has not had any External Funding

## References

[ref1] Adams T. Bezner J. and Steinhardt M. (1997) The conceptualization and measurement of perceived wellness: Integrating balance across and within dimensions. American Journal of Health Promotion. 11(3), pp.208–218. 10.4278/0890-1171-11.3.208 10165100

[ref2] Adams T. B. Bezner J. R. Drabbs M. E. Zambarano R. J. (2000) Conceptualization and measurement of the spiritual and psychological dimensions of wellness in a college population. Journal of American College Health. 48(4), pp.165–173. 10.1080/07448480009595692 10650734

[ref3] Allison D. G. (1997) Coping with stress in the principalship. Journal of Educational Administration. 35(1),39–55. 10.1108/09578239710156971

[ref4] Brett W. and Cathleen S. (2009) Wellness: It’s Impact on Student Grades and Implications for Business. Journal of Human Resources in Hospitality and Tourism. 8(2), pp.215–233. 10.1080/15332840802269858

[ref5] Chandler C. K. Holden J. M. and Kolander C. A. (1992) Counseling for spiritual wellness: Theory and practice. Journal of Counseling & Development. 71(2), pp.168–175. 10.1002/j.1556-6676.1992.tb02193.x

[ref6] De Klerk J. J. (2005) Spirituality, meaning in life, and work wellness: A research agenda. International Journal of Organizational Analysis. 13(1), pp.64–89. https://psycnet.apa.org/doi/10.1108/eb028998 (Accessed: 16 May 2020).

[ref7] Delle Fave A. and Massimini F. (2003) Optimal experience in work and leisure among teachers and physicians: Individual and bio‐cultural implications. Leisure Studies. 22(4), pp.323–342. 10.1080/02614360310001594122

[ref8] Diener E. Napa Scollon C. and Lucas R. E.,(2009) The Evolving Concept of Subjective Well-Being: The Multifaceted Nature of Happiness.In: Diener E. (eds) Assessing Well-Being. Social Indicators Research Series. vol 39. 10.1007/978-90-481-2354-4_4

[ref9] Diener E. and Seligman M. E. P. (2004) Beyond Money: Toward an Economy of Well-Being. Psychological Science in the Public Interest. 5(1), pp.1–31. 10.1111/j.0963-7214.2004.00501001.x (Accessed: 9 May 2020). 26158992

[ref10] Dorn F. J. (1992) Occupational wellness: The integration of career identity and personal identity. Journal of Counseling & Development. 71(2), pp.176–178. 10.1002/j.1556-6676.1992.tb02194.x

[ref11] Fylkesnes K. and Førde O. H. (1991) The Tromsø study: predictors of self-evaluated health-has society adopted the expanded health concept? Social Science & Medicine. 32(2), pp.141–146. 10.1016/0277-9536(91)90053-F 2014409

[ref12] Ghiselli R. F. La Lopa J. M. and Bai B. (2001) Job satisfaction, life satisfaction, and turnover intent: Among food-service managers. Cornell Hotel and Restaurant Administration Quarterly. 42(2), pp.28–37. https://doi.org/10.1177%2F0010880401422002

[ref13] Grad F. P. (2002). The preamble of the constitution of the World Health Organization. Bulletin of the World Health Organization. 80, pp.981–984. https://apps.who.int/iris/handle/10665/71722 (Accessed: 15 May 2020). 12571728 PMC2567708

[ref14] Guyatt G. H. Feeny D. H. and Patrick D. L. (1993) Measuring health-related quality of life. Annals of Internal Medicine. 118(8), pp.622–629. 10.7326/0003-4819-118-8-199304150-00009 8452328

[ref15] Hale C. J. Hannum J. W. and Espelage D. L. (2005) Social support and physical health: The importance of belonging. Journal of American College Health. 53(6), pp.276–284. 10.3200/JACH.53.6.276-284 15900991

[ref16] Hattie J. A. Myers J. E. and Sweeney T. J. (2004) A factor structure of wellness: Theory, assessment, analysis, and practice. Journal of Counselling & Development. 82(3), pp.354–364. 10.1002/j.1556-6678.2004.tb00321.x

[ref17] Hettler B. (1984) Wellness: encouraging a lifetime pursuit of excellence. Health Values. 8(4), pp.13–17. https://europepmc.org/article/med/10267293 (Accessed: 9 May 2020). 10267293

[ref18] Kitko C. T. (2001) Dimensions of wellness and the health matters program at Penn State. Home Health Care Management & Practice. 13(4), pp.308–311. https://doi.org/10.1177%2F108482230101300416

[ref19] Lengfelder J. R. (1987) Leisure wellness and time management: Is there a connection? College Student Journal. 21, pp.180–183. https://psycnet.apa.org/record/1988-31330-001 (Accessed: 9 May 2020).

[ref20] Morgeson F. P. and Humphrey S. E. (2006) The Work Design Questionnaire (WDQ): developing and validating a comprehensive measure for assessing job design and the nature of work. Journal of Applied Psychology. 91(6), pp.1321. https://psycnet.apa.org/doi/10.1037/0021-9010.91.6.1321 (Accessed 16 May 2020). 17100487 10.1037/0021-9010.91.6.1321

[ref21] Myers J. E. and Williard K. (2003) Integrating spirituality into counsellor preparation: A developmental, wellness approach. Counselling and Values. 47(2), pp.142–155. 10.1002/j.2161-007X.2003.tb00231.x

[ref22] O’Doherty D. Dromey M. Lougheed J. Hannigan A. (2018) Barriers and solutions to online learning in medical education - an integrative review. BMC Medical Education. 18(1), pp.130. 10.1186/s12909-018-1240-0 29880045 PMC5992716

[ref23] Roslender R. Stevenson J. and Kahn H. (2006) Employee wellness as intellectual capital: an accounting perspective. Journal of Human Resource Costing & Accounting. 10(1), pp.48–64. 10.1108/14013380610672675

[ref24] Slavin S. J. Schindler D. L. and Chibnall J. T. (2014) Medical student mental health 3.0: improving student wellness through curricular changes. Academic Medicine. 89(4), pp.573. 10.1097/ACM.0000000000000166 24556765 PMC4885556

[ref25] Swarbrick M. (2006) A wellness approach. Psychiatric Rehabilitation Journal. 29(4), pp.311. 10.2975/29.2006.311.314 16689042

[ref26] Ureda J. and Yates S. (2005) A systems view of health promotion. Journal of Health and Human Services Administration.pp.5–38. https://www.jstor.org/stable/41288055. (Accessed: 17 May 2020). 16521613

