# Supplementary material for: Fostering health professional students’ wellbeing during COVID -19 lockdown
Source: MedEdPublish (2016). 2020 Nov 6;9:248. [Version 1] doi: 10.15694/mep.2020.000248.1 (PMC10697555; doi:10.15694/mep.2020.000248.1)
Supplement: Peer review history

# Reviewer report: Reviewer response for version 1

**DOI:** 10.21956/mep.20100.r30979

**Published:** 2020-11-10

**Authors:** Samar Aboulsoud

**Affiliations:** 1 CAIRO UNIVERSITY, SCHOOL OF MEDICINE

This review has been migrated. The reviewer awarded 4 stars out of 5 Thank you for inviting me to review this interesting article.Students wellbeing and their ability to psychologically and physically cope with the new learning environment due to the COVID 19 is a very important subject. While we, as medical educators, focus on the learning aspects, curriculum delivery, and assessment techniques that are currently implemented, students’ wellbeing and emotional support strategies are frequently overlooked at these unprecedented and busy times.Inviting mentors to pay more attention to students’ wellbeing and proactively provide advice, resources and solutions that can help address the emerging challenges encountered by students is of utmost importance. The ideas of meditation, yoga and approaches that stimulate positive thinking are all great. The notion of addressing technical challenges, especially in remote areas with no or unstable internet connections, is very important and crucial. Technical difficulties can be very frustrating for students and I personally faced this challenging situation with some of my students.In conclusion the article brings some very interesting ideas. It will be useful to share the authors’ experience on practically applying those concepts as well as the perspectives of the students and how they received, adapted and benefited from the suggested mentorship techniques.Minor edit: the abstract is repeated twice.

Reviewer Expertise:

NA

No decision status is available

# Reviewer report: Reviewer response for version 1

**DOI:** 10.21956/mep.20100.r30978

**Published:** 2020-11-07

**Authors:** P Ravi Shankar

**Affiliations:** 1 American International Medical University

This review has been migrated. The reviewer awarded 4 stars out of 5 Thank you for the invitation to review this interesting, well-written and well-presented paper. The coronavirus disease-19 (COVID-19) has had a major impact on teaching-learning and well-being. Economic activity has been impacted and the pandemic has been active for longer than expected. In April-May, the expectation was that the pandemic will be short lasting and things may come back to normal by the end of this year at the latest. The pandemic has prolonged adding to the stresses and disruptions. Uncertainty has increased exponentially. The authors provide an overview of different aspects of well-being and how these can be maintained during the pandemic. The challenges of India are high population densities and crowded housing and living conditions which may create challenges in studying at home. The authors can briefly describe the steps taken at their institution to maintain student well-being. They mention about students staying in remote areas who have challenges in accessing learning. It will be interesting to know how the institution is supporting these students. I assume the institution must have admitted a new cohort of student recently. How are these students being oriented to the institution, the curriculum and the course? The abstract has been repeated and this can be corrected. All educators may find this article to be of interest.

Reviewer Expertise:

NA

No decision status is available
